# Impact of a Novel W2027L Mutation and Non-Target Site Resistance on Acetyl-CoA Carboxylase-Inhibiting Herbicides in a French *Lolium multiflorum* Population

**DOI:** 10.3390/genes12111838

**Published:** 2021-11-21

**Authors:** Shiv Shankhar Kaundun, Joe Downes, Lucy Victoria Jackson, Sarah-Jane Hutchings, Eddie Mcindoe

**Affiliations:** Herbicide Bioscience, Jealott’s Hill International Research Centre, Syngenta, Bracknell RG42 6EY, UK; joe.downes@syngenta.com (J.D.); lvjackson17@hotmail.co.uk (L.V.J.); sarah-jane.hutchings@syngenta.com (S.-J.H.); eddie.mcindoe@syngenta.com (E.M.)

**Keywords:** herbicide, acetyl-CoA carboxylase, *Lolium multiflorum*, mechanism of resistance, I1781L, I2041T, D2078G and W2027L target-site mutations, non-target site resistance, dPACS assay

## Abstract

Herbicides that inhibit acetyl-CoA carboxylase (ACCase) are among the few remaining options for the post-emergence control of *Lolium* species in small grain cereal crops. Here, we determined the mechanism of resistance to ACCase herbicides in a *Lolium multiflorum* population (HGR) from France. A combined biological and molecular approach detected a novel W2027L ACCase mutation that affects aryloxyphenoxypropionate (FOP) but not cyclohexanedione (DIM) or phenylpyraxoline (DEN) subclasses of ACCase herbicides. Both the wild-type tryptophan and mutant leucine 2027-ACCase alleles could be positively detected in a single DNA-based-derived polymorphic amplified cleaved sequence (dPACS) assay that contained the targeted PCR product and a cocktail of two discriminating restriction enzymes. Additionally, we identified three well-characterised I1781L, I2041T, and D2078G ACCase target site resistance mutations as well as non-target site resistance in HGR. The non-target site component endowed high levels of resistance to FOP herbicides whilst partially impacting on the efficacy of pinoxaden and cycloxydim. This study adequately assessed the contribution of the W2027L mutation and non-target site mechanism in conferring resistance to ACCase herbicides in HGR. It also highlights the versatility and robustness of the dPACS method to simultaneously identify different resistance-causing alleles at a single ACCase codon.

## 1. Introduction

Italian rye grass, *Lolium multiflorum* Lam., is an annual or biennial winter weed native to temperate Europe, north-west Africa, and south-west Asia [1]. It is a diploid (2x = 2n = 14) species and obligate outbreeder grown widely within and outside its native range as cover crop, forage, and turf [2,3]. Several varieties are available commercially including some high-yielding tetraploid cultivars [4]. *L. multiflorum* is characterised by a high genetic diversity and phenotypic plasticity [5]. It is a very adaptable species and prolific seed producer that can invade natural grassland and other plant communities that are subject to frequent disturbance [6]. It is also an important weed of arable crops and can cause significant yield loss if not properly managed [7]. In field trials carried out in the UK, for example, as few as five plants per metre square could reduce wheat yield by as much as 5% [8]. *L. multiflorum* can be effectively controlled by different herbicide modes of action including inhibitors of acetolactate synthase (ALS), acetyl-CoA carboxylase (ACCase), photosystem I/II, 5-enolpyruvylshikimate 3-phosphate (*EPSP*) synthase, very long chain fatty acid, and microtubule assembly [9].

ACCase-inhibiting herbicides, of interest in this study, can be grouped into aryloxyphenoxypropionates (FOPs), cyclohexanediones (DIMs), and phenylpyraxoline (DEN) based on their chemical structures [10]. They target monomeric chloroplastic ACCase of most grass weeds whilst having little to no activity on heteromeric plastidic ACCase of broadleaf species [11]. ACCase herbicides inhibit the carboxylation of acetyl-CoA into malonyl-CoA in sensitive grass weeds [12]. Rapid necrosis and plant death ensue due to depletion of de novo fatty acid synthesis, which are essential constituents of cell membranes [13]. Co-crystallography studies have shown subtle but important differences in the precise binding of FOP, DIM, and DEN herbicides to the carboxyl transferase (CT) domain of ACCase [14,15,16]. Some ACCase-inhibiting herbicides, such as clodinafop-propargyl and pinoxaden, provide selective control of grass weeds in certain monocotyledonous crops when co-applied with a safener [17].

Eighteen different ACCase inhibitor herbicides were developed over a span of 33 years, starting with diclofop-methyl in 1975, for the selective control of grass weeds in a number of cropping systems worldwide [10]. However, the overreliance of this mode of action, often as the single method of grass weed control, has resulted in resistance evolution in 49 different grass weed species [18]. As with other herbicide modes of action, resistance to ACCase-inhibiting herbicides can be classified into target site and non-target site mechanisms [19]. Target site resistance results from subtle changes in amino acid residues that are important for the proper binding of the herbicide to ACCase. Seven different ACCase codons at positions 1781, 1999, 2027, 2041, 2078, 2088, and 2096 (*Alopecurus myosuroides* equivalent) have been implicated in target site resistance [20]. Yeast-gene replacement assays and detailed whole plant dose response experiments have shown that the level of resistance depends on specific amino acid changes, ACCase-inhibitor, weed species, and the number of alleles in individual plants [21]. For instance, the W1999C mutation was found to confer resistance to fenoxaprop-ethyl but not to clodinafop-propargyl and haloxyfop-methyl [22]. Similarly, sethoxydim was affected when used on individuals that were homozygous for the W1999S mutation while still effective at controlling plants that were at the heterozygous state for this mutation [23]. Non-target site resistance to ACCase herbicides is basically due to the enhanced ability of plants to metabolise the toxophore before it reaches its target [24]. Resistance to ACCase herbicides due to metabolism is more prevalent than target site resistance but much less understood, with only a handful of causative cytochrome P450 and glutathione-*S*-transferase identified so far [20]. Metabolic resistance to ACCase-inhibiting herbicides is often multigenic, complex, and unpredictable [25]. It can sometimes endow resistance to different modes of action following an initial selection with an ACCase herbicide [26,27]. Metabolism-based resistance to ACCase herbicides has also been documented following grass weed exposure to herbicides that target acetolactate synthase [28].

The first case of resistance to ACCase herbicides in *L. multiflorum* was detected as early as 1987 in Oregon, USA [18]. To date, resistance to ACCase herbicides in the species is present in several other countries including the UK, France, Argentina, and Italy [18]. Although resistance to ACCase-inhibiting herbicides in *L. multiflorum* is a growing problem, a large number of populations are still effectively controlled by this herbicide’s mode of action when combined with other chemical and non-chemical tactics. Therefore, a better understanding of the mechanism of resistance to ACCase-inhibiting herbicides is important for developing weed management strategies that will ensure the long-term sustainability of this mode of action. In this study, we employed a holistic, biological, and molecular approach to investigate the mechanisms of resistance to ACCase-inhibiting herbicides in a French *L. multiflorum* population (HGR).

## 2. Materials and Methods

### 2.1. Seed Samples

**Figure 1:**
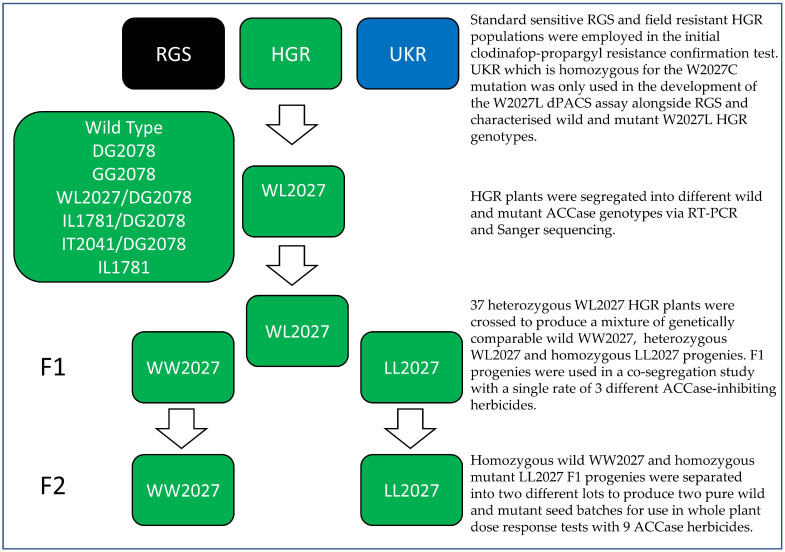
*L. multiflorum* populations and characterised genotypes used in this study.

Seeds from a *L. multiflorum* population (HGR) that had survived a clodinafop-propargyl application were collected in 2014 from a wheat field in Lafitte-Vigordane, Haute-Garone, France (Figure 1). A standard sensitive population (RGS) was sourced from Herbiseed (Twyford, UK) and used for comparison in all experiments. Additionally, a UK *L. multiflorum* population (UKR) that is homozygous for the W2027C mutation was employed alongside wild (WW2027) and mutant (WL2027 and LL2027) HGR genotypes in the development of the dPACS 2027-ACCase assay (Figure 1).

### 2.2. Confirmation of Resistance to Clodinafop-Propargyl

Around 50 seeds of HGR and RGS were sown in 12-cm pots containing a mixture of peat and compost in a 1:1 ratio. The pots were watered, fertilised, and kept in controlled glasshouse conditions set at 24 °C/16 h day, 18 °C night, 65% relative humidity, and a photon flux density of approximately 250 μmol quanta m^–2^ s^–1^. When the plants were at the two-leaf stage, they were treated with 0, 30, 60, and 120 g ai ha^−1^ of a clodinafop-propargyl in a spray cabinet mounted with a single mobile Teejet flat fan nozzle (11002VS) calibrated to deliver 200 L ha^−1^ at 200 kPa. Three replicate pots were used per population. The potted plants were assessed for percentage visual damage compared to the untreated control three weeks after the clodinafop-propargyl treatment.

### 2.3. Mechanism of Resistance Studies

#### 2.3.1. Analysis of ACCase for Target Site Resistance Mutations

A one-centimetre leaf fragment was collected from 96 HGR and 24 RGS untreated plants for sequencing the carboxyltransferase binding domain of *ACCase*. The RT-PCR and sequencing procedures were the same as described previously [29].

#### 2.3.2. Production of Wild WW2027 and Mutant LL2027 Seed Batches to Evaluate the Importance of the W2027L Mutation and Non-Target Site Resistance on ACCase-Herbicide Efficacy

HGR seeds were sown in a rectangular tray containing a mixture of peat and compost in a 1:1 ratio. After emergence, 600 seedlings were transplanted in individual 7.6-cm pots and genotyped as described in Section 2.2. Thirty-seven plants that were heterozygous for the W2027L change, but not containing any other known ACCase resistance mutations present in HGR, were separated and allowed to cross freely to produce F1 progenies (Figure 1). The observed genotype frequencies were compared to those expected under the null hypothesis of a 1:2:1 segregation ratio using a straightforward chi-square test on 2 degrees of freedom.

In total, 384 F1 plants were produced in individual pots and segregated into three lots of homozygous wild WW2027-HGR, homozygous mutant LL2027-HGR, and heterozygous WL2027-HGR plants as described above. Seventy-three homozygous wild and 80 homozygous mutant F1 individuals were transplanted in two different enclosed polytunnels and permitted to inter-breed to yield two pure homozygous WW2027-HGR and LL2027-HGR seed batches (Figure 1). The F1 progenies were employed to investigate plant survivorship to a single recommended rate of three different ACCase herbicides. The F2 homozygous wild and mutant seed lots were used in whole plant dose response tests to determine the level of resistance conferred by the W2027L target-site mutation and NTSR to a wide range of ACCase-inhibiting herbicides.

#### 2.3.3. Development of a dPACS Assay for Genotyping the W2027L Mutation

A PCR-RFLP-based-derived Polymorphic Amplified Cleaved Sequence assay (dPACS) was developed for the positive identification of the wild-type tryptophan and mutant leucine alleles in *Lolium multiflorum* plants [30].

##### Assay Design

**Figure 2:**
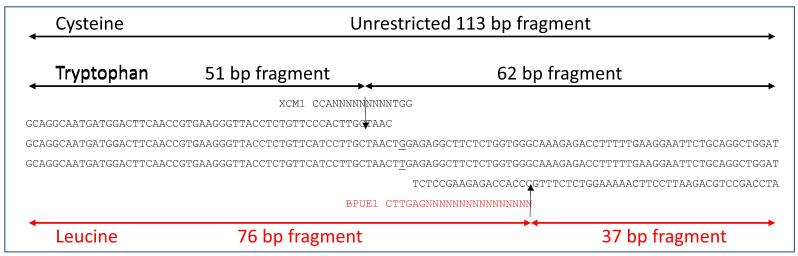
2027-ACCase-derived Polymorphic Amplified Cleaved Sequence assay: primer sequence, discriminating restriction enzymes, and expected PCR-RFLP profiles. The wild-type tryptophan allele will show two fragments of 51 and 62 base pairs and the mutant leucine allele will be manifested by two fragments of 76 and 37 base pairs. A mutant cysteine allele will result in an unrestricted band of 113 base pairs.

Characteristic of the dPACS procedure, the primers encompassed the whole region to be amplified except for the three nucleotides of the 2027 codon. The primers and restriction enzymes were selected using the dPACS freeware (http://opendata.syngenta.agroknow.com/models/dpacs, accessed on 3 July 2021). Both forward and reverse primers were 55 bp in length and the diagnostic restriction enzymes were *Xcm*I and *BpuE*I for the wild-type tryptophan and mutant leucine alleles, respectively (Figure 2). The forward primer (5′GCAGGCAATGATGGACTTCAACCGTGAAGGGTTACCTCTGTTCCCACTTGCTAAC3′) required three forced nucleotide changes at N-11, N-12, and N-13 (underlined) with respect to the second variable base of the 2027 codon to allow digestion with *Xcm*I (recognition site CCANNNNNNNNNTGG) and to positively identify the wild-type tryptophan allele (TGG triplet). The reverse primer (5′ ATCCAGCCTGCAGAATTCCTTCAAAAAGGTCTCTTTGCCCACCAGAGAAGCCTCT 3′) completely matched the template ACCase DNA. Restriction with *BpuE*I (recognition site CTTGAGNNNNNNNNNNNNNNNN) enzyme only occurs in the presence of the mutant leucine allele (TTG triplet). Digestion of the wild-type tryptophan allele with *Xcm*I would result in two shorter fragments of 51 and 62 base pairs whilst restriction of the mutant leucine allele with *BpuE*I would generate two fragments of 37 and 76 base pairs (Figure 2). The absence of either tryptophan or leucine allele at ACCase codon position 2027 would result in an undigested band of 113 bp.

##### PCR-RFLP and Gel Electrophoresis Procedure

Forty plants that were previously genotyped at codon position 2027 were used to develop the dPACS 2027 procedure. These consisted of 8 plants each of RGS (wild-type WW2027) and UKR (homozygous for the W2027C mutation) as well as WW2027-HGR, WL2027-HGR and LL2027-HGR progenies as produced in Section 2.3.2. One-centimetre leaf segments from each of the 40 plants were ground on a Spex Certiprep 2000 model Geno/Grinder (Metuchen, NJ, USA). DNA from the ground material was subsequently extracted on a KingFisher^TM^ Flex Purification platform (ThermoFisher Scientific, Waltham, MA, USA) using a Wizard Magnetic 96 DNA Plant System kit (Promega, Madison, WI, USA). puReTaq Ready-To-Go PCR beads (Amersham Biosciences, Chalfont St. Giles, UK) were employed to carry out polymerase chain reactions in a total volume of 25 µL containing 0.8 µM of each primer and about 50 ng of genomic DNA. The PCRs were run on an Eppendorf Master Cycle Gradient Thermocycler Model 96 programmed for an initial denaturation step at 94 °C for 2 min followed by 30 cycles of 30 s at 94 °C, 30 s at 60 °C, and 1 min at 72 °C. A final extension step for 10 min at 72 °C was also included.

Ten microlitres of neat PCR product were digested with a mixture of 5 units each of *Xcm*I and *BpuE*I (New England Biolabs, Hertfordshire, UK) in a total volume of 30 µL according to the manufacturer’s recommendations (NEB CutSmart buffer; incubation at 37 °C for 1 h) and analysed on 4% MetaPhor^TM^ agarose gel (Lonza, Walkersville, MD, USA) containing 0.5 µg mL^−1^ ethidium bromide run for 1 h at 70V with 1× TBE buffer.

#### 2.3.4. Co-Segregation Studies to Assess the Importance of the W2027L Target Site Mutation and NTSR on the Efficacy of Three ACCase Herbicides

The impact of the tryptophan to leucine mutation at ACCase codon position 2027 and NTSR in HGR was further investigated on a representative FOP, DIM, and DEN herbicide, namely fluazifop-P-butyl, cycloxydim, and pinoxaden, respectively. In total, 312 F1 progenies originating from a cross between heterozygous WL2027-HGR plants and 96 RGS were grown to the two-leaf stage in individual 7.6-cm pots in soil containing a mixture of peat and compost in a 1:1 ratio. The pots were watered, fertilised as required, and kept in controlled glasshouse conditions set at 24 °C/16 h day, 18 °C night, 65% relative humidity, and a photon flux density of approximately 250 μmol quanta m^–2^ s^–1^. Ninety-six F1 progenies and 24 RGS plants each were randomly chosen and treated with the recommended field rates of fluazifop-P-butyl (200 g ai/ha), cycloxydim (200 g ai/ha), and pinoxaden (60 g ai/ha). Twenty-four F1 progenies and 24 RGS plants were left untreated for comparison. Prior to the ACCase herbicide treatment, a 1-cm leaf tissue, each from the 312 progenies, was sampled and characterised at the 2027 ACCase codon position with the dPACS assay as described in Section 2.3.3 above. Survivorship to the three herbicides was assessed 21 days after treatment. Plant survival data from the co-segregation experiment were arranged as a 2 × 2 contingency table for each genotype comparison and analysed using Fisher’s Exact test. A *p*-value of less than 0.05 indicates a statistically significant result at the 5% probability level and provides evidence that the true levels of survivorship in the two treatments in question are genuinely different. The analysis was carried out using SAS software, version 9.4.

#### 2.3.5. Whole Plant Dose–Response Tests to Assess the Level of Resistance Conferred by the W2027L Mutation and NTSR

The pure homozygous wild WW2027 and mutant LL2027 seed batches produced in Section 2.3.2 were used in a whole plant dose–response experiment to determine the level of resistance conferred by the W2027L target-site mutation resistance to a wide range of ACCase herbicides. The standard sensitive population RGS was tested alongside the two former wild and mutant subpopulations to assess the degree of NTSR contained in HGR. Plants from the three populations were produced and maintained as described in Section 2.2. The rates for the nine different ACCase herbicides tested are provided in Table 1. The same herbicide doses were applied for all three plant groups employed except for diclofop-methyl and haloxyfop-methyl whereby split rates were used because of the very high levels of resistance endowed by the W2027L mutation and the good control of the standard sensitive RGS and WW2027-HGR samples at low rates of these two FOP herbicides.

Four replicate pots were tested for each herbicide rate. The pots were arranged in a randomised complete block design after herbicide application. Percentage visual biomass damage relative to untreated controls was assessed 21 days after treatment. The resulting percentages were analysed by straight line regression analysis of logit-transformed visual percent weed control against the logarithm of the rate applied. The logit transformation is given by Logit(P)= Ln[(P+12)/(100− P+12)], where P denotes visual percent damage and the addition of ½ to the numerator and denominator is required for assessments of 0% or 100%. Separate slopes were fitted to each genotype. GR50 estimates were obtained from the fitted regression lines and resistance indices were estimated as ratios of the respective GR50s. The data analysis was carried out using Syngenta’s proprietary software.

## 3. Results

### 3.1. Initial Clodinafop-Propargyl Resistance Confirmation Test

Clodinafop-propargyl applied at 0.5×, 1×, and 2× the recommended field rate completely killed the standard sensitive population RGS. In contrast, the HGR samples treated with the three herbicide doses were as healthy as untreated plants, suggesting high levels of clodinafop-propargyl resistance contained in the population.

### 3.2. Mechanism of Resistance to Clodinafop-Propargyl

#### 3.2.1. ACCase Analysis for Resistance Mutations

The RT-PCR procedure generated an expected 2272 DNA fragment for all the 96 HGR and 24 RGS plants tested. Analysis of the amplified DNA fragment showed around 97% homology with previously published ACCase carboxyl-transferase domain sequences, thus supporting the identity of the targeted gene. A large number of nucleotide changes was identified among the 120 plants sequenced. Most of these SNPs occurred at the 3rd base of codon triplets and amounted to synonymous changes. Eight non-synonymous changes resulting in R1844Q, D1864V, G1865V, R1870P, E1874A, N1878H, G1946E, and T2092R amino acid substitutions (numbering of the codon position is based on the closely related *A. myosuroides* species) were present in both RGS and HGR and therefore were not likely involved in conferring resistance to clodinafop-propargyl. Additionally, HGR contained four ACCase mutations that were not identified in RGS. These consisted of three known I1781L, I2041T, and D2078G ACCase resistant mutations and a novel W2027L change. The D2078G changes were present in 73 HGR individuals, of which 23 were homozygous GG2078, 35 were heterozygous DG2078, and eight, six, and one plants were double L1781/G2078, L2027/G2078, and T2041/G2078 heterozygotes, respectively. Four plants were heterozygous IL1781 and six plants were heterozygous WL2027. Importantly, 13 plants did not have any known ACCase mutations, suggesting that the HGR was also characterised by non-target site resistance to clodinafop-propargyl. As the impact of the I1781L, I2041T, and D2028G mutations in *L. multiflorum* or other grass weeds is well documented, this study focused on the novel W2027L variant and non-target site resistance contained in HGR [21,31].

#### 3.2.2. Development of a 2027-ACCase dPACS Assay

**Figure 3:**
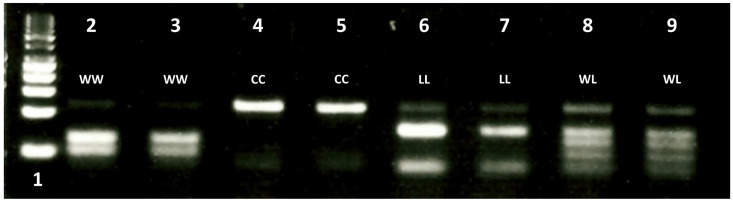
Characteristic dPACS profiles for wild and mutant 2027 ACCase alleles. Lane 1: 50 bp DNA ladder; lanes 2 and 3: wild type tryptophan allele (51/62 bp fragments), lanes 4 and 5: mutant cysteine alleles (unrestricted 113 bp fragment); lane 6 and 7: mutant leucine allele (37/76 bp fragments); lanes 8 and 9: heterozygous WL2027 (37/76 and 51/62 bp fragments).

A derived Polymorphic Cleaved Sequence (dPACS) assay was established for the cost-effective genotyping of resistance mutations at ACCase codon 2027 in *L. multiflorum* [30]. All three relevant known codon triplets at position 2027 were taken into consideration: namely, TGG, TTG, and TGT, which code for wild-type tryptophan and mutant leucine and mutant cysteine in the species. A large number of potential primer/enzyme combinations was generated by the dPACS software, including *Mfe*I and *Pvu*II with two forced mutations on the forward primer for detecting the wild-type tryptophan allele, and *Hind*III and *Taq*II for identifying the mutant leucine allele with two other mismatches on the forward and reverse primers, respectively. However, only one of these enzyme combinations, namely *Xcm*I and *BpuE*I, was non-interfering to allow the positive detection of the wild-type tryptophan and mutant leucine allele in a single PCR-RFLP assay and to generate different restriction profiles that could be unambiguously resolved on 4% MetaPhor^TM^ gel. Polymerase chain reaction generated an expected 113 bp fragment consisting of 110 bp of forward and reverse primers and three characteristic bases of the 2027 ACCase codon. Typical dPACS profiles as resolved on 4% MetaPhor^TM^ gel electrophoresis are provided on Figure 3.

The homozygous wild-type plants showed two shorter fragments of 51 and 62 base pairs upon restriction with *Xcm*I contained in the enzyme mix. Plants that were homozygous for the mutant leucine allele produced two smaller fragments of 37 and 76 bases pairs. As expected, heterozygous WL2027 plants displayed all four 51/62 and 37/76 restricted DNA fragments characteristic of the tryptophan and leucine alleles, respectively. The samples that were homozygous mutant CC2027 displayed the undigested 113 PCR fragment. The dPACS results completely matched the sequence analysis at codon 2027 for all 40 *L. multiflorum* plants, thus demonstrating the robustness of the assay developed in this study. It is noteworthy that low levels of undigested PCR fragments (characteristics of the PCR-RFLP approach), especially in lanes 6–9, should not be misinterpreted as a mutant cysteine allele as these are of much lower intensity than that the corresponding restricted bands.

#### 3.2.3. Impact of the W2027L Mutation and NTSR on Representative FOP, DIM, and DEN Herbicides

The F1 progenies employed to determine the effect of the W2027L mutation and non-target site resistance on fluazifop-P-butyl, cycloxydim, and pinoxaden segregated into a 0.90, 2.17, and 0.93 ratio for wild-type WW2027-HGR, heterozygous mutant WL2027-HGR, and homozygous LL2027-HGR genotypes. This conforms to an expected 1:2:1 ratio for a cross between heterozygous WL2027-HGR plants (χ = 0.363, *p* = 0.363). All standard sensitive RGS plants used for comparison were killed with the FOP, DIM, and DEN herbicides. Seventy-five percent WW2027-HGR, 98% WL2027-HGR, and 100% LL2027-HGR survived the fluazifop-butyl treatment (Table 2). Fisher’s exact test identified a significant difference in fluazifop-P-butyl survivorship between RGS and wild-type WW2027-HGR genotypes, suggesting that the FOP herbicide is impacted by NTSR contained in HGR. A significant difference in plant survival was also observed between heterozygous WL2027-HGR or homozygous mutant LL2027-HGR plants compared to wild-type WW2027-HGR individuals but not between WL2027-HGR and LL2027-HGR, implying dominance of the W2027L-ACCase mutation in conferring resistance to fluazifop-P-butyl. Four out of 19 WW2027-HGR plants survived the cycloxydim application with some evidence (*p* = 0.0314) that NTSR in HGR partially affects the DIM herbicide. On the other hand, there was no difference in survivorship between WW2027-HGR and WL2027-HGR or LL2027-HGR, indicating that the W2027L amino acid change does not impact on the efficacy of cycloxydim. NTSR contained in HGR also affected pinoxaden as 8 out of 24 WW2027-HGR plants survived the DEN treatment. There was no significant difference in plant survivorship between wild-type WW2027-HGR genotypes and WL2027-HGR or between wild-type WW2027-HGR and mutant LL2027-HGR, suggesting that the W2027L-ACCase mutation does not endow resistance to pinoxaden. The higher number of WL2027-HGR survivors compared to that of LL2027-HGR for pinoxaden was very probably due to an uneven number of WL2027-HGR heterozygotes and LL2027-HGR homozygotes containing NTSR among the randomly chosen F1 genotypes.

#### 3.2.4. Level of Resistance Conferred by the W2027L Mutation and Non-Target Site Resistance

**Figure 4:**
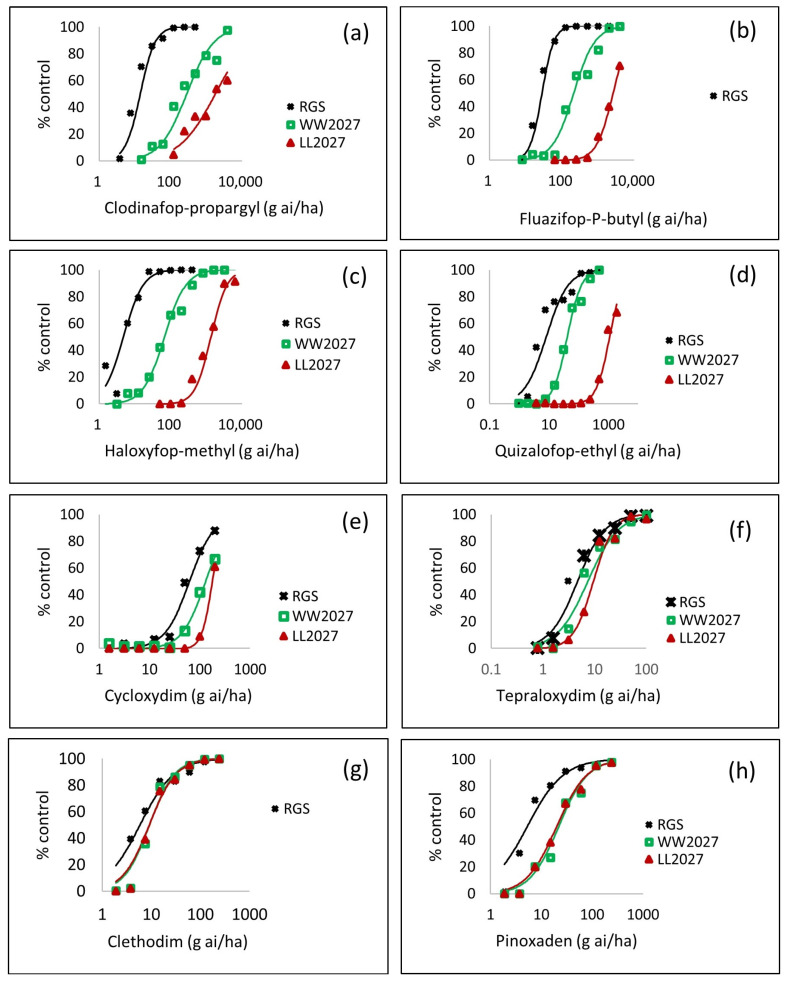
Whole plant dose–response test on three genotypes (RGS: standard sensitive, WW2027: homozygous wild type tryptophan, LL: homozygous mutant leucine and eight different ACCase-inhibiting herbicides: (**a**) clodinafop-propargyl, (**b**) fluazifop-P-butyl, (**c**) haloxyfop-methyl, (**d**) quizalofop-ethyl, (**e**) cycloxydim, (**f**) tepraloxydim, (**g**) clethodim, and (**h**) pinoxaden. The values plotted were back-transformed following analysis of logit transformed responses.

In order to evaluate the precise levels of resistance conferred by the novel W2027L target site resistance and NTSR in HGR to a wide range of ACCase-inhibiting herbicides, a series of whole plant dose response tests was carried out using pure wild-type WW2027-HGR and mutant LL2027-HGR seed batches alongside the standard sensitive population RGS. Comparison between wild-type WW2027-HGR and LL2027-HGR on the one side and wild-type WW2027-HGR and RGS on the other side allowed assessment of the level of resistance associated with the W2027L mutation and NTSR, respectively. A dose–response curve was generated for all nine FOP, DIM and DEN herbicides tested except for diclofop-methyl on the homozygous mutant LL2027-HGR, which was only controlled at 13% at the highest rate of 16 kg ai/ha. Nonetheless, a clear shift was observed between WW2027-HGR and LL2027-HGR, suggesting that W2027L target site mutation impacted on the efficacy of diclofop-methyl (Table 3). High levels of NTSR (RI = 37.6) in HGR also affected the efficacy of diclofop-methyl. The efficacy of clodinafop-propargyl was impacted by NTSR (RI = 19.9), contributing to three times more resistance than the W2027L mutation (RI = 6.3) (Figure 4, Table 3). The three other FOP herbicides tested, namely fluazifop-P-butyl, haloxyfop-methyl, and quizalofop-ethyl, were less impacted by NTSR than the W2027L target site mutation. Cycloxydim, tepraloxydim, clethodim, and pinoxaden were not affected by the W2027L mutation as the resistance indices ranged from 0.91–1.36 only (and confidence interval containing the value 1) when contrasting the WW2027-HGR with the LL2027-HGR genotypes. NTSR contained in HGR did not impact on tepraloxydim and clethodim while a small but significant shift was observed for pinoxaden (RI = 4.1) and cycloxydim (RI = 2.1), confirming earlier co-segregation results using a single rate of the DIM and DEN herbicides. When the W2027L target and non-target site mechanisms were combined (as assessed by comparing the LL2027 with RGS genotypes), the resistance indices ranged from 1.54 for clethodim to as high as 279.6 for haloxyfop-methyl through to 3.8 for pinoxaden and 92.7 for fluazifop-P-butyl.

## 4. Discussion

We successfully confirmed resistance and determined the mechanism involved in a French *L. multiflorum* population (HGR). Four different ACCase target site-resistant mutations were detected including the D2078G allele present in 76% of HGR plants. Previous studies based on yeast-gene replacement and enzyme assays, and whole plant dose response tests in *L. multiflorum*, *A. myosuroides*, *Phalaris minor*, *Eleusine indica*, and *Beckmannia syzigachne* have shown that the D2078G mutation confers broad resistance to all ACCase-inhibiting herbicides [22,29,32,33,34,35]. Although the glycine-2078 variant is characterised by pleiotropy, it has a definite fitness advantage under ACCase herbicide selection pressure and was the predominant target site resistance mechanism in a survey of *L. multiflorum* from the UK [36,37]. HGR also contained the I1781L mutation, which is documented to endow dominant resistance to all ACCase herbicides except clethodim for which control of the I1781L variant can be dose dependent [21]. At the Australian recommended field rate, for example, clethodim effectively killed heterozygous IL1781 *L. rigidum* plants whilst homozygous mutant LL1781 individuals survived the clethodim treatment [38]. The I1781L mutation was first identified in a *Setaria viridis* population in 2000 [39]. It was subsequently found in *Lolium* spp., *A. myosuroides*, *A. fatua*, *Phalaris minor*, *Leptochloa chinienis*, and *Echinochloa crus-galli* populations that have evolved resistance to ACCase-inhibiting herbicides [40,41,42,43,44,45]. The I1781L is not affected by a fitness penalty, explaining why the mutation is widely encountered in resistant grass weed populations [36,46]. The L11781 allele is also fixed in *Poa annua* and *Festuca rubra*, accounting for the intrinsic tolerance of these two species to ACCase-inhibiting herbicides [47,48]. The third known ACCase mutation in HGR consists of the rare I2041T change, which has so far been identified in *Alopecurus aequalis* only [29]. The T2041 allele was found to endow high and low levels of resistance to FOPs and DEN, respectively, whilst being sensitive to DIM herbicides.

Our study further detected a novel W2027L ACCase variant that warranted a detailed analysis to clearly establish its cross-resistance profile with a range of relevant ACCase herbicides. Contrary to most studies that use parental grass weed populations often containing a mixture of resistance mechanisms, F1 and F2 progenies segregating for the W2027L mutation were produced to adequately assess the importance of this novel variant on ACCase herbicide efficacy. The leucine 2027 allele conferred dominant resistance to fluazifop-P-butyl whilst not impacting on the efficacy of cycloxydim and pinoxaden in an initial study. Extensive whole plant dose response tests showed that the leucine 2027 allele conferred high levels of resistance to all five FOPs tested whilst the three DIM and DEN herbicides were not impacted by the mutation. The difference between FOP on the one side and DIM/DEN on the other side may be explained by the slightly different binding modes of ACCase-inhibiting herbicides to the carboxyltransferase domain. Co-crystallography studies using yeast as a surrogate species have shown that the anchoring of FOPs near the active site of the enzyme requires a large conformational change at the interface of the ACCase dimer [15]. On the other hand, DIMs and DEN probe a rather different region of the ACCase dimer and necessitate small but crucial conformational changes for their binding [14,16]. Whilst yeast ACCase crystal structures in complex with FOP, DIM, and DEN herbicides have revealed that tryptophan 2027 does not come into direct contact with ACCase-inhibiting herbicides, molecular docking and molecular dynamic simulations using homology models from *Setaria italica* and *A. myosuroides* have indicated that a mutation at this position will result in conformational changes that will impact the proper binding of FOP herbicides to the target enzyme [49]. In particular, a mutation at position 2027 will indirectly disrupt critical pi–pi interactions between FOP herbicides and two important phenylalanine and tyrosine residues, allowing the ACCase enzyme to function adequately and 2027-mutated grass weed populations to survive a FOP herbicide application.

It is noteworthy that a cysteine variant at ACCase codon 2027 was also implicated in resistance to ACCase herbicides in several grass weed species, including *Lolium* spp., *A. myosuroides*, *A. fatua*, and *L. chinenis* to name but a few [41,42,50,51]. As with the leucine allele, the non-conserved cysteine substitution conferred high levels of resistance to FOPs whilst DIM and DEN herbicides were impacted slightly based on yeast-gene replacement assays [22]. Sensitivity of the C2027 mutant to DIMs and resistance to FOPs was also observed at the whole plant level [51]. The response to pinoxaden was more nuanced depending on the species and the populations employed [52,53]. A low to moderate level of resistance to pinoxaden was sometimes observed, very probably due to additional underlying mechanisms that were not always taken into consideration in some of the studies.

Given the importance of the 2027 amino acid residue for sensitivity to FOP herbicides, a derived Cleaved Amplified Polymorphic Sequence (dCAPS) assay was developed for genotyping grass weeds at this ACCase codon position [54]. The dCAPS was designed to positively identify the wild-type tryptophan allele via restriction with *Xmn*I whilst a mutation at the 2027 position would result in an undigested PCR band. Here, we took advantage of the versatility of the dPACS approach to provide an alternative assay that could positively identify both the tryptophan and leucine in a single assay [30]. With the proposed 2027 dPACS method, a cysteine appears as a non-restricted band, thus allowing clear differentiation of all three different tryptophan, cysteine, and leucine alleles at codon position 2027. A similar dPACS strategy was recently employed to develop highly transferable and robust markers to positively identify as many as four critical amino acid residues at EPSPS codon 106 using a single PCR product and different discriminating enzymes [30]. The dPACS approach appears to be the method of choice for addressing multiple allelic variants that cause target site resistance to herbicides in weeds [55].

HGR was also characterised by NTSR as is most commonly the case with grass weeds that have evolved resistance to ACCase-inhibiting herbicides [21]. A notable exception to this observation is a *L. multiflorum* from the UK, which was found to contain the C2088R target site mutation only [56]. The level of NTSR in HGR was very much compound dependent. The cereal-safe FOP herbicide diclofop-methyl and clodinafop-propargyl were the most affected followed by the less metabolisable FOP herbicides haloxyfop-methyl fluazifop-butyl, and quizalofop-ethyl. On the other hand, clethodim and tepraloxydim were not impacted, similar to what has been observed so far for grass weed populations containing NTSR [21,57]. Pinoxaden, which is also cereal-safe, was partially affected, with 33% of wild-type WW2027-HGR plants surviving the treatment. Whist most previous studies have shown that pinoxaden was effective at controlling non-target site resistance selected by cereal-selective FOP herbicides, unsatisfactory pinoxaden control of blackgrass populations that did not have a mutated target has also been documented [29,58]. More recently, pinoxaden metabolism mediated by a cypP450 gene has been unravelled in a *L. multiflorum* population from Australia [59]. CypP450-based metabolism to pinoxaden has also been identified in an *Echinochloa phyllopogon* population with no previous history of ACCase herbicide use [28]. Cross-resistance to pinoxaden was endowed by genes selected following repeated use of the ALS-inhibiting herbicide bensulfuron-methyl in the *E. phyllopogon* population. Interestingly, cycloxydim at the recommended field rate failed to control around 21% of wild-type HGR individuals. Whilst a shift in DIMs efficacy on NTSR in *L. multiflorum* was observed previously, the herbicide did control the population when applied at the recommended field rate [23,29]. Most DIM herbicides, including cycloxydim, are not cereal-safe and are only used for the control of grass weeds in dicotyledonous break crops. Due to its robustness to metabolism, cycloxydim and other DIMs, such as sethoxydim, have often been employed as an indicator of FOP-DIM-DEN target site mutation in grass weed populations that have not been controlled with DIM herbicides [29,51,60]. Our finding tends to indicate that NTSR to cycloxydim is gradually being selected in *L. multiflorum* and therefore a lack of grass weed control by the herbicide may not always be indicative of a target site resistance mutation. A build-up of non-target site resistance to cycloxydim is very likely operating in blackgrass populations in the UK as well since DIMs are commonly applied for the post-emergence control of the species in rotational oilseed rape and sugar beet crops in the country. This is corroborated with a recent survey of black-grass populations for DIM resistance-causing mutations and the level of control with cycloxydim [61]. Despite an overall good correlation between the frequency of DIM mutations and efficacy of cycloxydim, there was a number of surviving individuals that could not be accounted for by the target site-based mechanism [61]. The potential evolution of metabolic resistance to cycloxydim merits further investigation at the physiological and genetic level as it may reveal an unconventional route to DIM metabolism in grass weeds that is not present in small grain cereal crops.

## 5. Conclusions

Our study identified as many as four different target site resistant mutations, including a novel W2027L FOP-specific variant and NTSR that also affect cycloxydim in a French *L. multiflorum* population. The presence of multiple resistance mechanisms in a single population is testimony of the large genetic variability and high potential for resistance evolution that characterises obligate cross-pollinated species, such as *L. multiflorum*. A similar observation was made for single Australian and European *Lolium* spp. Populations, which contained several target site-resistant mutations and NTSR following extensive selection with ACCase-inhibiting herbicides [38,41]. The insight gained from this study will not only be useful for effectively managing the *L. multiflorum* population with alternative herbicidal and non-herbicidal strategies but will also be valuable for designing the next generation of herbicides that can overcome the types of resistance contained in the population.

## 6. Patents

This section is not mandatory but may be added if there are patents resulting from the work reported in this manuscript.

## Figures and Tables

**Table 1:** ACCase-inhibiting herbicides and rates employed in the whole plant dose–response study.

Herbicide	Rates Applied in g ai/ha
Clodinafop-propargyl	3.8, 7.5, 15, 30, 60, 120, 240, 480
Diclofop-methyl	15, 31.25, 62.5, 125, 250, 500, 1000, 2000, 4000, 8000, 16,000
Fluazifop-P-butyl	7.8, 15, 31,25, 62.5, 125, 250, 500, 1000
Haloxyfop-methyl	1.5, 3.125, 6.25, 12.5, 25, 50, 100, 200, 400
Quizalofop-ethyl	0.93, 1.875, 3.75, 7.5, 15, 30, 60, 120
Cycloxydim	3.125, 6.25, 12.5, 25, 50, 100, 200, 400
Tepraloxydim	6.25, 12.5, 25, 50, 100, 200, 400, 800
Clethodim	1.9, 3.8, 7.5, 15, 30, 60, 120, 240
Pinoxaden	1.875, 3.75, 7.5, 15, 30, 60, 120, 240

**Table 2:** Correlation between plant genotypes at ACCase codon 2027 and survivorship at single rates of three ACCase-inhibiting herbicides.

Herbicide	Rate (g/ha)	Comparison	No. of Survivors/Total		*p*-Value (2-Sided)
			Genotype 1	Genotype 2	
Fluazifop-P-butyl	200	WW2027 v WL2027	18/24	51/53	0.0097
		WW2027 v LL2027	18/24	19/19	0.0265
		WL2027 v LL2027	51/53	19/19	1
		RGS v WW2027	0/24	18/24	<0.00001
Cycloxydim	200	WW2027 v WL2027	4/19	13/55	1
		WW2027 v LL2027	4/19	8/22	0.3246
		WL2027 v LL2027	13/55	8/22	0.2712
		RGS v WW2027	0/24	4/19	0.0314
Pinoxaden	60	WW2027 v WL2027	8/24	25/48	0.2093
		WW2027 v LL2027	8/24	4/24	0.3177
		WL2027 v LL2027	25/48	4/24	0.0049
		RGS v WW2027	0/24	8/24	0.0039

**Table 3:** Resistance indices estimated from the dose–response test on three genotypes: RGS (standard sensitive), WW2027 (homozygous wild-type tryptophan), and LL2027 (homozygous mutant leucine) and nine ACCase-inhibiting herbicides.

Herbicide	WW2027 vs. RGS	LL2027 vs. RGS	LL2027 vs. WW2027
Clodinafop-propargyl	19.9 (13.6–29.3)	125.7 (62.8–251.6)	6.3 (3.0–13.1)
Diclofop-methyl	37.6 (22.6–62.5)	>121.2	>3.2
Fluazifop-P-butyl	7.5 (5.6–10.1)	92.7 (63.0–136.6)	12.3 (8.1–18.6)
Haloxyfop-methyl	14.0 (9.5–20.6)	279.6 (191.3–408.5)	20.0 (14.5–27.5)
Quizalofop-ethyl	5.13 (3.30–7.97)	137.4 (83.5–226.0)	26.8 (18.0–39.9)
Cycloxydim	2.09 (1.31–3.33)	2.84 (2.00–4.03)	1.36 (0.88–2.08)
Tepraloxydim	1.58 (1.00–2.51)	2.03 (1.41–2.92)	1.28 (0.85–1.95)
Clethodim	1.56 (0.82–2.95)	1.54 (0.80–2.97)	0.99 (0.54–1.81)
Pinoxaden	4.17 (2.20–7.91)	3.80 (1.98–7.31)	0.91 (0.57–1.46)

## Data Availability

Any further inquiries regarding data generated as part of this publication can be directed to the corresponding author.
